# Aged garlic extract as a potential prophylactic to reduce the progression of endometriosis and associated pain burden

**DOI:** 10.3389/fpain.2022.1057830

**Published:** 2022-11-17

**Authors:** Emily Redwood, Virginie Lam, Ryusuke Takechi, Deborah Anne Kerr, Connie Jackaman, Arazu Sharif, John Charles Louis Mamo

**Affiliations:** ^1^ School of Population Health, Curtin University, Perth, WA, Australia; ^2^ Curtin Health Innovation Research Institute, Curtin University, Perth, WA, Australia; ^3^ Curtin Medical School, Curtin University, Perth, WA, Australia

**Keywords:** endometriosis, pain, nutraceuticals, prophylactic, aged garlic extract

## Abstract

Endometriosis is a complex and potentially debilitating condition that has major impact on quality of life. There is emerging evidence that biological compounds found in garlic (*Allium sativum*) may be effective for attenuating endometrial pain. Suggested mechanisms for efficacy include modulation of inflammation and potent antioxidant effects. Aged-garlic-extract (AGE) is a centuries old process describing ethanolic extracts of garlic bulbs for 12–20 months. The AGE formulation realised contains a complex array of stabilised biologics with significant immunomodulatory effects relevant to inflammatory conditions. This perspective article puts forward a hypothesis that AGE should be considered as a prophylactic to manage endometrial pain.

## Introduction

Endometriosis is a chronic systemic disease that is pathologically characterised by uterine tissue proliferating outside of the uterine cavity (1, 2). The extra-uterine growth commonly occurs on the pelvic peritoneum and can also grow on ovaries, in the rectovaginal septum and in rare cases, exist in the pericardium, pleura, or brain (1). Endometriosis is suggested to be estrogen-dependent, however the physiological mechanisms for this association are not fully understood (1).

The American Society for Reproductive Medicine classification for endometriosis is a globally adopted scoring system where points are allocated regarding lesion characteristics and grouped into stages I (minimal) to IV (severe) (3). Stage I generally includes few superficial implants, whereas stage IV includes multiple large, deep implants and dense adhesions (3). However, a higher visually appointed severity score does not correlate with pain or symptoms experienced *per se*, which often results in delayed diagnosis (3, 4).

It is estimated that endometriosis affects 170 million people globally and ∼10% of women of reproductive age (1, 5–7). Symptoms include chronic pelvic pain, dysmenorrhea, dyspareunia, resulting in psychological effects and impaired social function (4, 8). Depression and anxiety due to chronic pain and stress surrounding fertility may amplify the burden of disease (9). Presently, there are limited treatments available to reduce endometrial progression, or attenuate endometrial pain, with significant impact on quality of life (4).

## Aetiology and pathology

The prevailing paradigm for the aetiology of endometriosis is the theory of retrograde menstruation (1). It is proposed that uterine cells attach to distant sites from the peritoneal cavity due to the backflow of menstrual blood and cells (10). Women with endometriosis have an abnormally higher number of progenitor stem cells than women without the condition during menses (11), consistent with causality. Other pathways for disease progression include immune dysfunction, characterised by dysregulation of recruited leukocytes with limited capacity to clear fragments, increasing the propensity for lesion formation (1). Endometriotic lesions located outside the pelvis, in women without a uterus and indeed in men, indicate triggers other than retrograde menstruation (2). Coelomic metaplasia of mesothelial cells may also induce endometriosis (2).

The onset and progression of endometriosis is complex and multifactorial. Evidence suggests that either a single factor, or a combination of factors such as inflammation, oxidative stress, tumour promoting-genes, or endocrine modulation cell proliferation, microvascular tone, and angiogenesis, may be highly relevant (2, 12).

**Figure 1:**
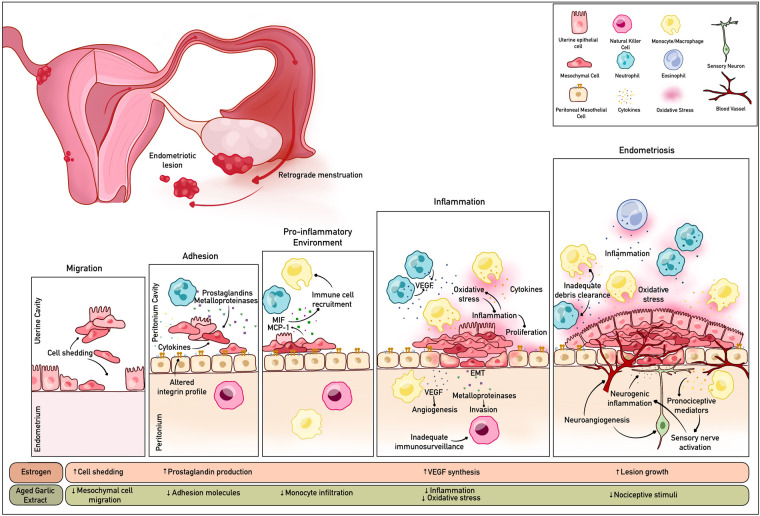
Schematic summary of the cellular disruption pathways, inflammation and oxidative stress associated with endometriosis. The bioactive compounds in AGE may modulate these mechanisms by targeting various pathways. Created with BioRender.com.

Garcia-Gomez et al. describes that inflammation is central to the pathophysiology of endometriosis and the symptoms of pain and infertility (13). However, there is complexity in delineating how immune cell function exacerbates, or attenuates endometrial disease progression. Macrophage migration inhibitory factor and monocyte chemoattractant protein-1 attracts monocytes/macrophages into endometriosis lesions (14). Inflammatory cytokines, prostaglandins and metalloproteinases are increased in endometriosis and may promote ectopic adhesion (12). Inflammatory mediators trigger epithelial-mesenchymal transition resulting in a cyclical inflammatory loop as a consequence of increased synthesis of vascular endothelial growth factor (VEGF) and angiogenesis (12, 15). Neutrophils have the ability to synthesise and secrete VEGF (16). The accumulation of these leukocytes in endometriosis has been proposed to be crucial for early disease progression due to the pro-angiogenic and inflammatory modulation effects (17, 18). Increased integrin secretion allows for ectopic implantation and concomitant hyperestrogenism can amplify lesion growth and development (12). Inadequate immunosurveillance may also exacerbate endometrial tissue proliferation. There is reduced cytotoxicity of natural killer cells in endometriosis patients which may be attributed to poor clearance of ectopic endometriotic cells (19). The dysregulation of the aforementioned pathways resulting in inflammation, invasion and metastasis could be considered as targets for endometriosis treatment (refer to Figure 1).

## Endometrial pain treatment

The pain mechanisms associated with endometriosis are suggested to be positively associated with inflammation and angiogenesis resulting in neurogenic inflammation and cross-organ sensitization (20). Treatments for endometriosis are presently limited. Contemporary opportunities include surgical intervention, chronic use of nonsteroidal anti-inflammatory drugs, oral contraceptives, or endocrine modulation (gonadotrophin-releasing hormone agonists) (21). Surgical intervention can provide transient pain relief, however, recurrence within 5 years occurs in up to 50% of patients (22). Pharmacological interventions have limited efficacy and may have significant off-target effects including; osteoporosis, lipid profile changes and metrorrhagia (21).

In recent years nutraceuticals have gained increased attention as an alternative for endometriosis related pain relief. Bioactive compounds naturally occurring in plants have emerging evidence for anti-endometriotic effects, including quercetin, curcumin, resveratrol and naringenin (21). It has been suggested that garlic has anti-inflammatory and antioxidant effects that may be relevant in the context of endometriosis. A recent study by Amirsalari et al. found that consumption of 400 mg of garlic powder results in a significant reduction of pain symptoms in women with endometriosis (23). They suggest that garlic can reduce oxidative stress, prostaglandin production, limit proliferation of endometrial cells, and improve estrogen elimination (23). While this is the first human trial known using garlic as a nutraceutical for endometriosis, the results are consistent with a substantial body of literature describing the anti-inflammatory and antinociceptive effects of garlic.

Kim et al. found that in an *in vitro* model of activated endometrial stromal cells, vascular adhesion molecule-1 and intercellular adhesion molecule-1 expression was reduced in response to 500 ml hexane-extract of aged black garlic (HEABG) (24). This study reported that HEABG resulted in a reduction in the proliferation of endometrial stromal cells that were activated by tumour necrosis factor (TNF)-α (24). The biological elements of garlic may also be important in attenuating the damaging effects of oxidative stress associated with inflammation. Infertile women with endometriosis were found to have lower levels of peritoneal fluid and serum antioxidant markers (total antioxidant capacity, catalase enzyme and superoxide dismutase enzyme) content compared to infertile women without endometriosis (25).

Garlic is rich in N-acetylcysteine (NAC) which has been reported to have anti-proliferative effects in endometriomas. With 3 months of NAC supplementation, taken orally, mean diameter of lesions was reduced by 1.5 mm whereas those without treatment had a significant increase in lesion size diameter of 6.6 mm (26). These effects were attributed to the downregulatory effects of NAC on inflammatory pathways and inhibiting cell migration (26).

In humans, antinociceptive effects of garlic have been indicated in patients with knee osteoarthritis which has similar inflammatory sequelae to endometriosis with increased inflammatory factors and associated TNF-α (27–29). Similar findings have been reported in rheumatoid arthritis, which is subject to increased oxidative stress, and peripheral arterial occlusive disease (30, 31). It has been suggested that pain reduction may be directly attributable to the antioxidant effect of garlic (32). In animal studies there have been a number of induced models of pain demonstrating an attenuation with the provision of garlic derivatives (33). In murine models with central and peripheral induced pain, there was increased antinociception through the provision of garlic shoot extract (34).

AGE is processed by chopping raw garlic cloves and storing them in 15%–20% ethanol for up to 20 months (35). The main organosulphur compounds in AGE are water-soluble *S*-allyl-cysteine (SAC), allin, ɣ-glutamylcysteines, ɣ-glutamyl-S-allylcystine, and *S*-allylmercaptocysteine (SAMC) (32). The SAC and SAMC concentrations in AGE are estimated to be >60-fold more abundant per unit weight than in raw garlic due to the ageing process (36–38), enabling a pharmacological dose to be tested more readily clinically. These specific compounds have exceedingly potent immunomodulatory and antioxidant properties that may be relevant to endometriosis progression and pain management. Phenolic compounds in AGE such as allicin also contribute to antioxidant activity (36).

## Conclusion

Endometriosis can result in a high burden of disability and is costly to treat. Prophylactive therapies that delay progression and/or decrease endometrial pain are a global health priority. A body of literature suggests that *Allium sativum* and particularly, ethanolic extracts of the garlic bulb containing enriched stabilised biologicals may be effective in attenuating endometrial progression and pain burden.

## Data Availability

The original contributions presented in the study are included in the article/Supplementary Material, further inquiries can be directed to the corresponding author/s.
